# Interleukin-10 Induces Expression of Neuroendocrine Markers and PDL1 in Prostate Cancer Cells

**DOI:** 10.1155/2020/5305306

**Published:** 2020-07-31

**Authors:** Abrar Samiea, Jeff S. J. Yoon, Christopher J. Ong, Amina Zoubeidi, Thomas C. Chamberlain, Alice L.-F. Mui

**Affiliations:** ^1^Immunity and Infection Research Centre, Vancouver Coastal Health Research Institute, Vancouver, Canada; ^2^Department of Surgery, University of British Columbia, Vancouver, Canada; ^3^Department of Biochemistry and Molecular Biology, University of British Columbia, Vancouver, Canada; ^4^Vancouver Prostate Centre and Department of Urologic Sciences, University of British Columbia, Vancouver, BC, Canada

## Abstract

Interleukin-10 (IL10) is best studied for its inhibitory action on immune cells and ability to suppress an antitumour immune response. But IL10 also exerts direct effects on nonimmune cells such as prostate cancer epithelial cells. Elevated serum levels of IL10 observed in prostate and other cancer patients are associated with poor prognosis. After first-line androgen-deprivation therapy, prostate cancer patients are treated with androgen receptor antagonists such as enzalutamide to inhibit androgen-dependent prostate cancer cell growth. However, development of resistance inevitably occurs and this is associated with tumour differentiation to more aggressive forms such as a neuroendocrine phenotype characterized by expression of neuron specific enolase and synaptophysin. We found that treatment of prostate cancer cell lines *in vitro* with IL10 or enzalutamide induced markers of neuroendocrine differentiation and inhibited androgen receptor reporter activity. Both also upregulated the levels of PDL1, which could promote tumour survival *in vivo* through its interaction with the immune cell inhibitory receptor PD1 to suppress antitumour immunity. These findings suggest that IL10's direct action on prostate cancer cells could contribute to prostate cancer progression independent of IL10's suppression of host immune cells.

## 1. Introduction

Prostate cancer (PCa) is among the leading causes of cancer mortality worldwide. At early stages, PCa proliferation is mostly androgen-dependent [1–4]; thus, PCa cells are initially treated with androgen-deprivation therapy (ADT) [2, 5–8]. Once tumours develop androgen-independent growth, patients are treated with AR pathway inhibitors (ARPI) such as enzalutamide (ENZ). While advanced PCa is initially controlled with hormonal therapies targeting the androgen receptor (AR) pathway, recurrence occurs due to emergence of ENZ resistant, lethal castration-resistant PCa (CRPC). Autopsy series suggest that up to 25% of CRPC patients are resistant to ARPI, shed their dependence on the AR, and exhibit a continuum of features associated with the neuroendocrine (NE) lineage [9].

Notably, the NE phenotype can be enhanced by factors in the tumour environment such as cytokines like interleukin-6 (IL6) [10]. The action of IL6 on PCa cells has been extensively studied [11], and IL6 receptor signalling has been reported to induce NE differentiation through different mechanisms including its canonical activation of STAT3 transcription factor [12]. Another cytokine that signals through STAT3 is interleukin-10 (IL10). In fact, both IL10 and IL6 have been reported to be excessively expressed in metastatic androgen-independent PCa cells [13] and serum levels of IL10 and IL6 are elevated in patients resistant to ENZ treatment compared to sensitive patients [14]. These observations suggest that both cytokines may contribute to the development of more aggressive tumours with NE phenotype [15, 16].

IL10 is best studied as an anti-inflammatory, immune suppressive cytokine [17–19] that contributes to promoting cancer aggressiveness by acting on immune cells to suppress the antitumour immune response [20]. IL10 serum levels in cancer patients correlate with poor prognosis in prostate cancer patients [21] and are positively correlated with Gleason scores [22]. IL10 could be produced either by the tumour cells themselves [13, 23–25] or by tumour elicitation of tumour-infiltrating, IL10 producing immune cells [26, 27]. IL10 inhibition of the antitumour immune response includes suppression of myeloid (macrophage and dendritic cell) and *T* effector cell function [27–30]. IL10 also upregulates expression of PDL1 (CD274) on myeloid cells [31]. PDL1 binds to the inhibitory receptor PD1 on T cells resulting in inactivation of the T cell and inhibition of the host T cell antitumour immune response [32, 33].

However, in the early 2000s, Stearns et al. reported that IL10 also has direct actions on PCa cells [34–36]. IL10 treatment of PCa cell lines increased TIMP1 [34] and decreased MMP1 and MMP2 synthesis [35]. How the IL10 regulation of TIMP1 and MMP1/MMP2 expression contributes to PCa progression is not clear, but elevated TIMPs and MMPs are associated with higher grade PCa [37]. No work has been done regarding the direct effect of IL10 on PCa since the studies published by the Stearns group, but we became interested in the direct actions of IL10 on PCa cells because of the interesting observations reported by Bishop et al. [16] regarding PDL1 expression in cells from patients who are ENZ resistant.

Bishop et al. found that, in tumour biopsies from ENZ resistant patients, PDL1 is predominantly increased on the PCa cells rather than in tumour immune infiltrating cells [16]. This prompted us to examine whether IL10 directly induces expression of NE-associated proteins and PDL1 on PCa cells *in vitro*. We compared the effect of IL10 with that of IL6 or ENZ treatment on different AR-dependent and AR-independent PCa cells. We also assessed the ability of both IL10 and IL6 to modulate AR activity in LNCaP cells expressing a stably transduced AR controlled GFP reporter [38]. We found that addition of IL10 to PCa cells *in vitro* promoted development of NE-like characteristics and enhanced the surface expression of PDL1 protein. This has implications for potential therapies involving the use of IL10 for the treatment of PCa.

## 2. Materials and Methods

### 2.1. Cells

The LNCaP prostate cancer cell line [39] was maintained in RPMI-1640 (HyClone, Logan, Utah) supplemented with 9% FBS (HyClone, Logan, Utah). LNCaP cells expressing ARR2PB-eGFP were kindly provided by Dr. Paul Rennie (Vancouver Prostate Centre, Vancouver, British Columbia). ENZ resistant and ENZ sensitive 42D^ENZR^ and 16D^CRPC^ CRPC cell lines, respectively, were kindly provided by Dr. Amina Zoubeidi (Vancouver Prostate Centre, Vancouver, British Columbia). These cells were generated from *in vivo* LNCaP xenografts described previously [15]. 16D^CRPC^ cells were maintained as the LNCaP cells. 42D^ENZR^ cells were maintained in 10 *µ*M ENZ, RPMI-1640, 9% FBS. All cells were kept in a 37°C, 5% CO2, 95% humidity incubator.

### 2.2. Reagents

Antibodies used include 1 : 1000 diluted EP4 (C-4) mouse antibody (Santa Cruz Biotechnology, Santa Barbara, CA), 1 : 1000 diluted neuron specific enolase (A-5) mouse antibody (NSE, Santa Cruz Biotechnology, Santa Barbara, CA), 1 : 500 diluted synaptophysin (H-8) mouse antibody (SYP, Santa Cruz Biotechnology, Santa Barbara, CA), 1 : 5000 GAPDH rabbit antibody (Sigma, Oakville, ON), 1 : 5 diluted PDL1 (MIH1) mouse antibody (BD Pharmingen, Canada), and 25 *µ*g/mL *F*_C_ block (BD Pharmingen, Canada). Human IL10 and IL6 were from StemCell Technologies (Vancouver, Canada). Enzalutamide (MDV 3100) was from Cayman Chemical Company (Ann Arbor, MI). IL10 and IL6 (StemCell Technologies) stocks were reconstituted in sterile water at 10,000 ng/mL as instructed by the manufacturer. The working solution was prepared in the growth medium and used at the indicated concentration. ENZ stock was dissolved in DMSO at 10,000 *µ*M concentration, and the working solution was prepared in the growth medium and used at a final concentration of 10 *µ*M.

### 2.3. Cell Stimulations

For western blot studies, cells were seeded at 3 × 10^4^ cells per well on 24-well tissue culture plates 1 day prior to start of treatments in growth medium supplemented with a 1% FBS for LNCaP cells or 5% FBS for 16D^CRPC^ or 42D^ENZR^ CRPC cells. Cells were then treated with media alone (untreated) or 100 ng/mL IL10, IL6, or 10 *µ*M ENZ stimulation solutions that were prepared using the growth medium for 7 days. For the flow cytometry experiments, cells were plated at 1 × 10^5^ cells per well on 6-well tissue culture plates using growth medium supplemented with a 1% FBS for LNCaP cells or 5% FBS for 16D^CRPC^ or 42D^ENZR^ CRPC cells 1 day prior to stimulation. Next day, cells were either untreated or treated with 50 ng/mL IL10, IL6, or 10 *µ*M ENZ for 7 days. For 42D^ENZR^ cells, ENZ was removed from the cell culture at time of plating to study the direct effect of different stimuli on ENZ resistant cells.

ARR2PB-eGFP cells were seeded in 1% FBS media for one passage to minimize GFP background expression. Cells were plated at 8 × 10^4^ cells per well on 24-well in 1% RPMI media overnight. Next day, cells were stimulated with media alone (untreated) or either 20 ng/mL IL10, IL6, or 10 *µ*M ENZ stimulation solutions for the required time points.

### 2.4. Immunoblot Analysis

Cells were rinsed with cold PBS and lysed with hot 2 x Laemmli sample buffer. Proteins were separated by 12.5 % SDS-PAGE, followed by electroblotting onto polyvinylidene fluoride (PVDF) membrane (Millipore, Etobicoke, ON). Membranes were blocked 1 hour with 3% bovine serum albumin in 20 mM Tris HCl/pH 7.5, 150 mM NaCl (TBS), rinsed with 0.05% Tween 20/TBS (TBST, wash buffer), and probed with primary antibodies prepared in 3% blocking buffer at room temperature overnight. The following day, membranes were washed 3 × 10 min, incubated 1 hour at room temperature with Alexa Fluor® 660 anti-mouse IgG or Alexa Fluor® 680 anti-rabbit IgG antibodies in TBST buffer (ThermoFisher, Nepean, ON), washed, and imaged using a LI-COR Odyssey Imager.

### 2.5. Flow Cytometry Analysis

For measurement of PDL1 surface expression, cells stimulated with IL10, IL6, ENZ, or media alone as described above were rinsed with cold PBS followed by the addition of 2 mM EDTA/PBS to each well for 5 min. 200 *µ*L of FACS buffer (3% FBS in PBS) was added and cells were collected and spun at 1000 x*g* for 5 min at 4°C. Cells were resuspended in 25 *µ*LL of 25 *µ*g/mL *F*_C_ block (BD-Pharmingen, Mississauga, Canada) in FACS buffer and transferred to a V-bottom 96-well plate for 15 min at 4°C. Anti-human PDL1 PE-conjugated antibody (BD-Pharmingen, Mississauga, Canada) was added for 1 hour. Cells were then washed with FACS buffer 3 times and analyzed (minimum 10 K events within the cell gate) on a Canto II (BD-Biosciences, Mississauga, Canada). The FACS data were analyzed with FlowJo_V10 (BD-Biosciences, Mississauga, Canada). Cells were gated based on forward scatter height (FSC-H) and side scatter height (SSC=H) pattern (“cell gate”), and the PE (FL2) fluorescence of cells within this cell gate was determined.

For the measurement of GFP positive expressing cells in ARR2PB-eGFP LNCaP, cells were collected at the indicated time points and were rinsed with cold PBS followed by the addition of 200 *µ*L 2 mM EDTA to each well to lift the cells. 200 *µ*L of FACS buffer was added and cells were spun at 1000 x*g*g for 10 min at 4°C. Cell pellets were resuspended in FACS buffer and data were acquired (5000 events within the cell gate) as described above. 16D^CRPC^ cells were used as a negative control to define the GFP negative population in the GFP channel (FL1).

### 2.6. Statistical Analysis

Quantification of band intensities in immunoblots was performed using LI-COR Odyssey imaging system and Image Studio™ Lite software (LI-COR Biosciences, Lincoln, NE). GraphPad Prism 6 (GraphPad Software Inc., La Jolla, CA) was used to perform all statistical analyses. Statistical details can be found in figure legends. Values are presented as means ± standard deviations. One-way ANOVA was performed where required with appropriate multiple comparisons tests. Differences were considered significant when *p* ≤ 0.05.

## 3. Results

We examined the effect of IL10 on PCa cell lines representing various stages of cancer development. LNCaP cells are an AR positive, androgen sensitive cell line derived from metastatic lymph node PCa tumours [39]. IL6 induction of LNCaP morphological alternations has been previously reported [10, 40], and ENZ treatment has been shown to induce neuroendocrine differentiation (NED) in the cells *in vitro* and *in vivo* [15]. The 42D^ENZR^ and 16D^CRPC^ cell lines both still express the AR and were derived from LNCaP xenografts that had been serially passaged in castrated mice, which were treated or not, respectively, with ENZ. The 42D^ENZR^ cell line is more resistant to ENZ and expresses more basal PDL1 than LNCaP and 16D^CRPC^ cells and represents an aggressive neuroendocrine phenotype [15]. The 16D^CRPC^ cell line represents an androgen-independent (CRPC), ENZ sensitive cell [15].

### 3.1. IL10 Induction of Morphological Transformation and NE Proteins Consistent with Neuroendocrine Differentiation

**Figure 1:**
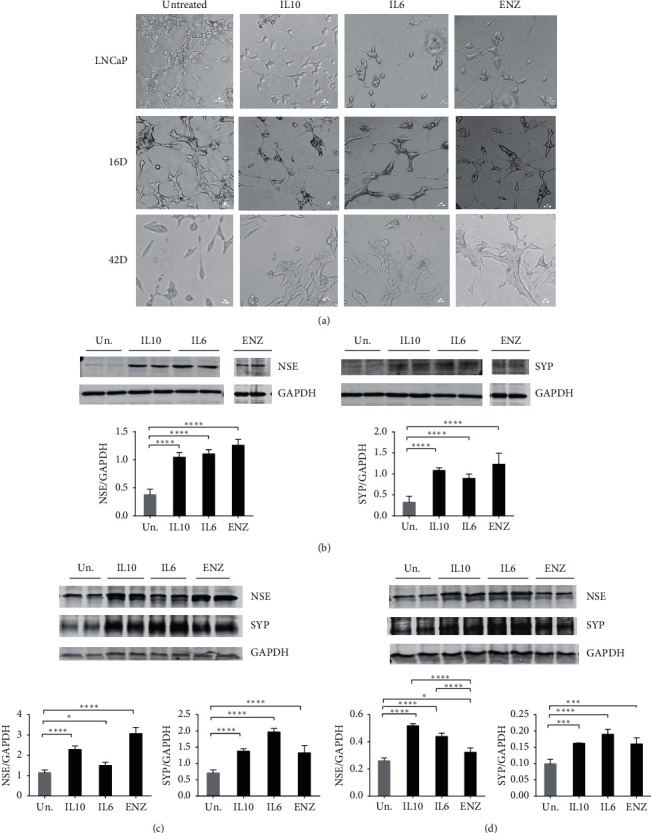
IL10 induction of morphological changes and expression of NSE and SYP neuroendocrine markers in PCa cells. PCa cells grown *in vitro* were stimulated with 100 ng/ml IL10, 100 ng/mL IL6, or 10 *µ*M ENZ for 7 days. The morphological changes induced by different treatments in LNCaP, 16D^CRPC^, and 42D^ENZR^ resistant cells were imaged using a light microscope (a). The expression levels of NSE or SYP were determined by immunoblotting of the cell lysates in LNCaP (b), 16D^CRPC^ (c), and 42D^ENZR^ resistant (d) cells. Data plotted represents NSE and SYP band intensities normalized to GAPDH protein levels. The statistical significance for the difference between untreated (Un.) and different treatment was determined by one-way ANOVA test with Tukey's correction. ^*∗∗∗∗*^*p* < 0.0001, ^*∗∗∗*^*p* < 0.001, ^*∗∗*^*p* < 0.01, and ^*∗*^*p* < 0.05. Data are representative of three independent experiments.

The NED phenotype is characterized by distinct morphological features [40]. We examined the effect of treating LNCaP cells with IL10, IL6, and ENZ for 7 days on cell morphology. IL10 treatment resulted in distinct morphological changes that appeared after 4 days and these alternations were more pronounced after 7 days of treatment (Figure 1(a)). The IL10 treated cells became long and branched and had neuritic-like extensions. These morphological changes were comparable to those induced by IL6 and ENZ (Figure 1(a)). We next examined the effect of IL10 on 16D^CRPC^ and 42D^ENZR^ cells. In the case of the 16D^CRPC^ cells, IL10, IL6, and ENZ treatment showed a phenotype similar to that observed in LNCaP cells (Figure 1(a)). To study effect of IL10 on 42D^ENZR^ cells, we first removed ENZ from their culture media for a day prior to the experiment. The following day, cells were treated with media, IL10, IL6, or ENZ. 7 days after treatment, the untreated cells were oblong in shape while the IL10, IL6, and ENZ treated cells are more planar (Figure 1(a)).

Next, we tested whether IL10 could induce expression of the neuronal markers, neuron specific enolase (NSE) or synaptophysin (SYP), which have been observed to be induced by IL6 or ENZ in PCa cells *in vitro* [15, 40]. As shown in Figure 1(b), NSE and SYP expression levels were significantly increased by IL10 in LNCaP cells compared to untreated cells. This increase of NSE and SYP levels was comparable to cells treated with IL6 or ENZ. These results show that both IL10 and IL6 may work similarly in androgen sensitive PCa cells, leading to a NED phenotype associated with the expression of NE proteins. In the ENZ sensitive cell 16D^CRPC^, IL10 and ENZ induced similar levels of NSE and SYP protein (Figure 1(c)). In contrast, IL6 treatment significantly upregulated SYP protein but modestly increased NSE protein expression (Figure 1(c)). The lower ability of IL6 to increase NSE levels has also been described in DU145 and C4-2 PCa cells [41]. As expected in the ENZ resistant 42D^ENZR^ cells, ENZ induction of NSE and SYP protein was weaker than in LNCaP and 16D^CRPC^ cells (Figure 1(d)). IL10 and IL6 treatment elevated NSE and SYP levels (Figure 1(d)) higher than that seen with ENZ, but lower than that observed in LNCaP and 16D^CRPC^ cells. These data are summarized in Table 1.

### 3.2. IL10-Induced Inhibition of AR Activation

**Figure 2:**
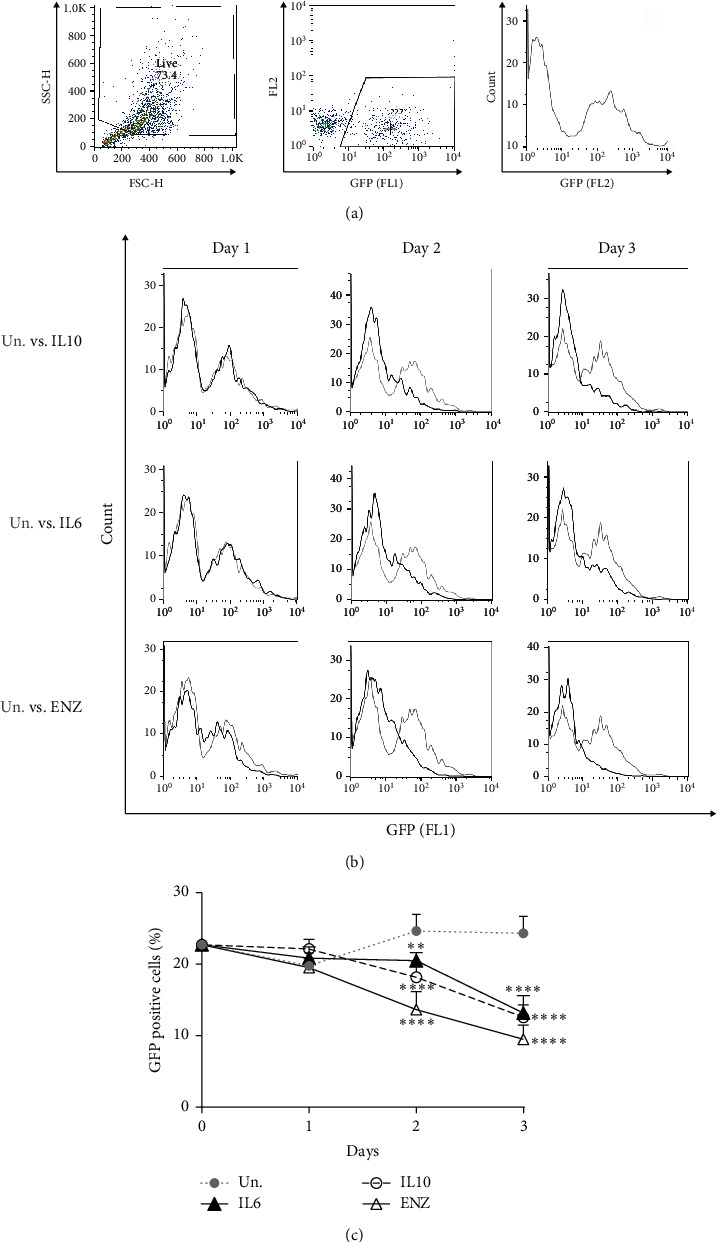
Inhibition of AR transactivation in IL10, IL6, and ENZ treated cells. LNCaP cells were either untreated (Un) or treated with 100 ng/mL IL10, IL6, or 10 *µ*M ENZ. Cells were collected for flow cytometry analysis at the indicated time points. (a) The gating strategy and how % GFP cells are determined. (b) Histograms are from one of three representative experiments. (c) The % GFP positive cells determined, as shown in panel (a), are plotted for the different treatment groups over time. The statistical significance for the difference between untreated and different treatments was determined by two-way ANOVA test with Tukey's correction. ^*∗∗∗∗*^*p* < 0.0001, ^*∗∗∗*^*p* < 0.001, ^*∗∗*^*p* < 0.01,and ^*∗*^*p* < 0.05; ns = not significant.

We next examined whether IL10 treatment might inhibit AR activity, in a LNCaP cell line stably expressing a GFP reporter under the control of the AR regulated probasin promoter (ARR_2_PB) [38]. IL10, IL6, and ENZ all inhibited GFP expression with similar kinetics in the LN-ARR2PB-EGFP cells (Figures 2(a) and 2(b)). However, the degree of AR activity inhibition seems to be the greatest by ENZ followed by IL10 and IL6 as shown in Figure 2(c). Whether IL10 and IL6 inhibition of AR activity is responsible for the acquisition of NE characteristics needs to be determined.

### 3.3. Effect of IL10, IL6, and ENZ on PDL1 Levels in PCa Cancer Cells

**Figure 3:**
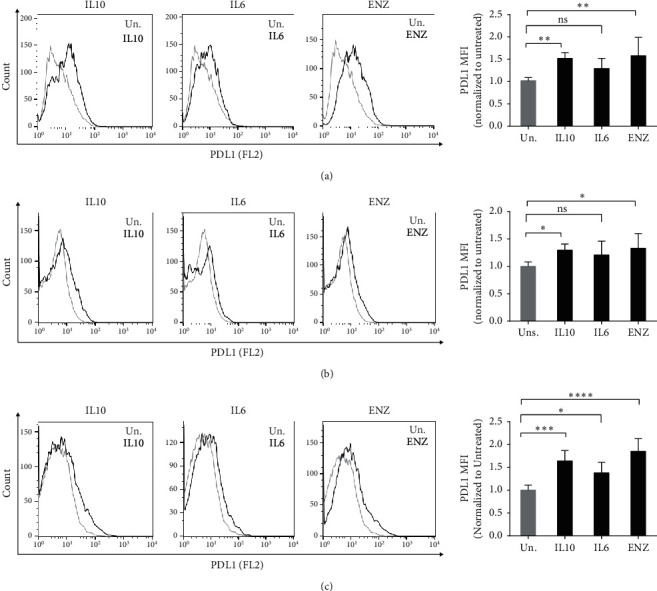
Effect of IL10, IL6, and ENZ on PDL1 levels in PCa cells. Surface expression of PDL1 after 7 days of treatment with 50 ng/mL IL10 or IL6 and 10 *µ*M ENZ in LNCaP (a), 16D^CRPC^ (b), and 42D^ENZR^ (c) cells. Histograms are shown here as representative from one of three experiments. Bar graphs show mean fold MFI changes from three independent experiments (MFI of untreated cells were normalized to 1, and MFI of the samples were normalized to untreated cells). Experiments were repeated three times. The means of the MFI fold change are plotted in panel (c). The statistical significance for the difference between untreated and different treatment was determined by one-way ANOVA with Tukey's correction. ^*∗*^*p* < 0.05, ^*∗∗∗*^*p* < 0.001, and ^*∗∗∗∗*^*p* < 0.0001; ns = not significant.

Recently, PDL1 protein levels were reported to be highly elevated in the PCa cells in tumour biopsies from enzalutamide resistant patients [16]. To determine whether IL10 or IL6 treatment can directly alter PDL1 protein expression in PCa cells, we measured PDL1 expression levels after 7 days of treatment with IL10, IL6, or ENZ using flow cytometry. As shown in Figure 3(a), IL10 and ENZ treatment of LNCaP cells increased PDL1 surface expression compared to untreated cells. IL6 treatment showed only a modest induction of PDL1 expression which was confirmed to be statistically insignificant. We also examined the effect of IL10, IL6, and ENZ on PDL1 levels in 16D^CRPC^ androgen sensitive cells and 42D^ENZR^ androgen resistant cells. In 16D^CRPC^ cells, we found that IL10 and ENZ but not IL6 treatment significantly increased PDL1 levels compared to untreated cells (Figure 3(b)) similar to that seen in the LNCaP cells. Finally, we examined the effect of IL10, IL6, and ENZ treatment on 42D^ENZR^ cells that had been cultured out of ENZ. IL10, IL6, and ENZ treatment all significantly upregulated PDL1 expression (Figure 3(c)).

### 3.4. Prostaglandin E Receptor EP4 Subtype (EP4) Is Marginally Upregulated upon IL10 Treatment in Different PCa Cells

**Figure 4:**
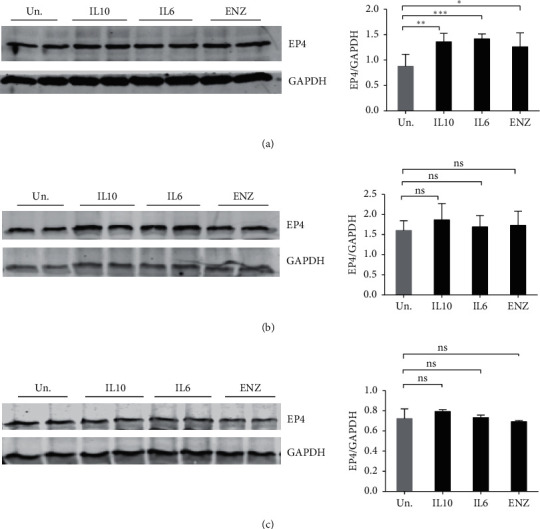
Slight upregulation of EP4 in IL10, IL6, and ENZ treated LNCaP cells. (a) LNCaP, (b) 16D^CRPC^, (c) 46D^ENZR^ resistant cells were stimulated with 100 ng/mL IL10, IL6, or ENZ for 7 days prior to lysate collection. Expression levels of EP4 were determined by immunoblotting of cell lysates. Data plotted represents EP4 band intensities normalized to GAPDH protein levels. Experiments were repeated 3 times. The statistical significance for the difference between untreated and different treatment was determined by one-way ANOVA with Tukey's corrections. ^*∗∗∗*^*p* < 0.001, ^*∗∗*^*p* < 0.01, and ^*∗*^*p* < 0.05; ns = not significant.

Autocrine/paracrine produced prostaglandin E2 (PGE2) binds to the prostaglandin E2 receptor 4 (EP4) and can support proliferation of PCa cells by stimulating PI3K/Akt and cAMP-dependent PKA pathways [42]. PGE2 has been reported to induce NED phenotype in PCa cells [43], and in our work with macrophages we found that IL10 induction of EP4 protein is required for IL10 action in these cells [44]. Thus, we examined whether IL10 or IL6 might upregulate EP4 expression to promote PGE2-induced NED. As shown in Figure 4(a), both IL10 and IL6 (and ENZ) treatment slightly increased EP4 levels in LNCaP cells. However, neither IL10, IL6, or ENZ induced EP4 protein levels in 16D^CRPC^ or 42D^ENZR^ cells after 7 days of treatment as compared to untreated cells (Figures 4(b) and 4(c)).

## 4. Discussion

A challenging aspect in treating prostate cancer is that even those patients who are treated with the new androgen receptor antagonists such as ENZ, after first-line therapy fails [45, 46], also develop resistance to these drugs [47–50]. Some recurrent and resistant tumours are associated with the development of the more aggressive NE phenotype [51] with 39% of tumours classified as either intermediate or pure neuroendocrine prostate cancer (NEPC) [52]. AR antagonist action results in appearance of NEPC tumour cells, but cytokines such as IL6 which are elevated in PCa patients can also directly induce NED [10] in LNCaP cells. We report here that IL10, another cytokine upregulated in PCa patients, can also induce NE-like characteristics.

We chose to study the LNCaP cell line and the 16D^CRPC^ or 42D^ENZR^ lines derived from castration and ENZ resistant *in vivo* LNCaP tumours, respectively, generated by Bishop et al. [16]. We chose these cells for two reasons. The first is that all the papers in the literature describing the effect of IL6 on prostate cancer cells used LNCaP cells [10, 12, 40, 53–56]). The second is that we are interested in the potential change in IL10 and IL6 responsiveness of a PCa cell as they become castration or enzalutamide resistant. Thus, a strength of our study is the examination of a classic PCa cell and *in vivo* derived derivatives that represent later stages of PCa, and this is the first demonstration of IL10 that behaves like IL6 on PCa cells. But one limitation to our study is that we only used these LNCaP related cells lines. The generalizability of our observations will require a survey of other PCa cell lines and of PCa tumour biopsies.

As summarized in Table 1, IL10 treatment leads to expression of NSE in LNCaP, 16D^CRPC^, and 42D^ENZR^ cells to levels similar to that induced by IL6. IL10, IL6, and ENZ treatment also increased SYP levels in all three cell lines, with the IL6-induced SYP levels in 16D^CRPC^ cells higher than that seen with either IL10 or ENZ. Future studies will determine the mechanisms underlying the increase of these NE markers. Both IL10 receptor (IL10R) [57] and IL6R [58] signalling involve the use of the STAT3 transcription factor, but pathways unique to each receptor have also been described. For instance, IL6R signalling includes activation of the MAPK cascade [10] in PCa cells. Perhaps the MAPK pathway contributes to the increased SYP expression induced by IL6 in the 16D^CRPC^ cells. On the other hand, IL10R signalling has mostly been studied in immune cells where the SHIP1 inositol phosphatase contributes to IL10 inhibition of macrophage activation [59, 60]. SHIP1 is expressed only in hemopoietic and immune cells, so signalling pathways downstream of the IL10R in epithelial cells, other than STAT3, remain to be characterized.

Since there is a strong inverse relationship between AR activity and the induction of NE-like characteristics [9, 15, 61–64], we looked at whether IL10 and IL6 inhibited AR activity. We tested the effect of IL10 and IL6 on AR activation using LNCaP cells expressing the AR reporter construct, ARR_2_PB-EGFP [38], and found both inhibited GFP expression within 2 days of treatment. Notably, the degree of IL6 and IL10 inhibition of AR activity was lower than ENZ treatment. ENZ directly binds to AR [65] and presumably inhibits AR activation by androgens in the media. The direct action of ENZ on the AR likely explains the more rapid and stronger effect of ENZ on ARR_2_PB promoter activity. The effect of IL6 is in agreement with previous reports [38, 66]. Jia et al. reported that IL6 inhibits AR-dependent expression of the androgen regulated PSA gene by preventing the recruitment of p300 coactivator to the PSA promoter and this inhibition was STAT3 dependent [66]. Whether IL10 signalling also inhibits coactivator recruitment remains to be determined.

In contrast, other investigators have concluded that IL6 stimulates AR activity in other experimental settings [67–70], where AR activity is assessed through transient transduction of cell lines with AR expressing vector and a reporter gene construct [67–70]. However, as discussed [38, 71], these transient transduction approaches may not accurately recapitulate physiological signalling, since they do not reflect the precise levels of the androgen receptor which can affect coactivator recruitment. Furthermore, AR expression can vary between different replicates of the same assay, depending on the health of the cells and the efficiency of the transduction. This can be a problematic factor since CRPC tumours have been shown to contain altered levels of AR and AR coactivators which reactivate AR signalling [72–74]. To avoid these complications, we used the LN-ARR2PB-EGFP cell line that stably expresses an AR-responsive GFP reporter construct [38].

We also examined whether IL10 or IL6 might upregulate the PGE2 receptor, EP4, to promote PGE2-induced NED. In our studies of IL10 action in macrophage cells, we found IL10 induction of EP4 protein expression is needed for IL10 inhibition of macrophage production of inflammatory cytokines [44]. In PCa cells, activation of EP4 by PGE2 has been reported to increase the expression of metastatic-related proteins [42]. EP4 upregulation of these proteins was mediated in a cAMP-dependent PKA dependent manner [42]. EP4 was also shown to be significantly upregulated during progression to castration resistance [75, 76]. Other reports also indicated the involvement of PGE2 [43], cAMP [10], and cAMP dependent kinase, particularly PKA [77], in promoting NE phenotype in PCa cells which are known to be mediated through EP4 receptor activation in PCa cells [42]. However, we found EP4 levels constitutively highly expressed, and IL10, IL6, or ENZ treatment only very slightly increased EP4 protein levels in LNCaP cells. No EP4 upregulation occurred in response to these agents in either 16D^CRPC^ or 42D^ENZR^ cells. These observations suggest that neither IL10 nor IL6 likely enhances NE differentiation through increasing EP4 protein levels.

We found that IL10, which is elevated in PCa patients, may directly act on some PCa cells to increase PDL1 expression (Table 1). IL10 and ENZ treatment increased PDL1 expression in all three PCa lines we tested. In contrast, IL6 showed slight upregulation of PDL1 only in 42D^ENZR^ cells. Our observation that exogenously added IL6 only weakly induces PDL1 in one of the three PCa cell lines we tested differs from the high expression of PDL1 that Xu et al. reported in C4-2 IL6 expressing cells [78]. However, Xu et al. had lentivirally transduced their C4-2 cells with IL6 and prolonged exposure to autocrine IL6 which likely improves PDL1 expression [78]. Prolonged exposure to IL6 may be needed to ensure proper glycosylation of PDL1. Chan et al. showed that, in hepatocellular carcinoma cells, IL6-activated JAK1 phosphorylates PDL1 at tyrosine Y112, which enhances the association of PDL1 with endoplasmic reticulum-associated (ER-associated) *N*-glycosyltransferase isoform STT3A [79]. STT3A is a catalytic subunit of the oligosaccharyltransferase complex that is needed for N-glycosylation and stabilization of PDL1 [79]. However, it is unclear whether N-glycosylation of PDL1 is needed for expression in PCa cells since IL10 and ENZ can both induce PDL1 levels even though IL6 cannot.

The widespread clinical use of more potent androgen receptor pathway inhibitor drugs, such as ENZ, to treat CRPC tumours has increased the emergence of more aggressive tumour types such as the NEPC [15, 80–83]. Immune checkpoint based immunotherapy approaches including anti-PDL1 therapy have been successful in other cancer types [84]. Unfortunately, clinical trials using anti-PDL1 were not successful in prostate cancer [85–87], and this was thought to be due of the lack of PDL1 expression on prostate cancer cells [87–89]. However, Bishop et al. [16] recently showed that ENZ LNCaP resistant tumours do express PDL1. Further studies are needed to determine if human ENZ resistant tumours upregulate PDL1 and if so, this subset of patients might benefit from anti-PDL1 treatment. Of note, Ihle et al. recently showed that, in bone metastatic PCa tumours, PDL1 is more highly expressed in PCa cells in blastic type lesions than the lytic lesions [90].

Another novel cancer immunotherapy being evaluated clinically involves the administration of IL10. IL10 is best studied for its inhibitory action on immune cells such as macrophages, but IL10 can also stimulate CD8^+^ T cell antitumour immunity and was tested in a clinical trial of multiple tumour types [91]. Naing et al. showed that IL10 treatment increased CD8^+^ activity and prolonged patient survival in some cancer types [91]. The combination of IL10 and anti-PDL1 also showed good responses [92] in other cancer patients. However, the use of IL10 needs to be carefully considered before using it on PCa patients because as we have shown in this study, IL10 can increase the NED phenotype in PCa cells.

In conclusion, our studies suggest that the role of cytokines in contributing to the NED of PCa tumours warrants further investigation. This includes examination of other PCa cell lines, primary PCa cells and in mouse tumour models. PCa tumours which have metastasized to the bone have been reported to be infiltrated by immune cells [90] which may be the source of IL6 and/or IL10. As pointed out by the authors, the interaction of PCa cells and the immune infiltrating cells should be examined.

## Figures and Tables

**Table 1:** Summary of NSE, SYP, and PDL1 expression in PCa cells treated with IL10, IL6, and ENZ. “++,” “+,” “+/-” indicate very strong, strong, and moderate expression, respectively, as compared to the untreated cells. “−” indicates no significant expression as compared to the untreated cells.

Cell line	NSE expression			SYP expression			PDL1 expression		
	IL10	IL6	ENZ	IL10	IL6	ENZ	IL10	IL6	ENZ
LNCaP	+	+	+	+	+	+	+	−	+
16DCRPC	+	+	+	+	++	+	+	−	+
42DENZR	+/−	+/−	+/−	+/−	+/−	+/−	+	+/−	+

## Data Availability

The data that support the findings of this study are available from the corresponding author upon reasonable request.
